# Polyethylenimine-Modified *Bombyx mori* Silk Fibroin as a Delivery Carrier of the ING4-IL-24 Coexpression Plasmid

**DOI:** 10.3390/polym13203592

**Published:** 2021-10-19

**Authors:** Longxing Niu, Guo Chen, Yanfei Feng, Xueping Liu, Peng Pan, Linling Huang, Ying Guo, Mingzhong Li

**Affiliations:** National Engineering Laboratory for Modern Silk, Department of Textile Engineering, College of Textile and Clothing Engineering, Soochow University, Suzhou 215123, China; niu18860902006@163.com (L.N.); chenguo148@126.com (G.C.); fengyanfei0123@126.com (Y.F.); 18862107248@163.com (X.L.); ppanpanpeng@stu.suda.edu.cn (P.P.); hll4146@163.com (L.H.); gyguoyingy@163.com (Y.G.)

**Keywords:** silk fibroin, gene carrier, lung cancer, inhibitor of growth 4, interleukin-24

## Abstract

One of the major challenges for lung cancer gene therapy is to find a gene delivery vector with high efficiency and low toxicity. In this study, low-molecular-weight polyethyleneimine (PEI, 1.8 kDa) was grafted onto the side chains of *Bombyx mori* silk fibroin (BSF) to prepare cationized BSF (CBSF), which was used to package the plasmid DNA (pDNA) encoded by the inhibitor of growth 4 (ING4) and interleukin-24 (IL-24). FTIR and ^1^H-NMR spectra demonstrated that PEI was effectively coupled to the side chains of BSF by amino bonds. The results of the trinitrobenzene sulfonic acid method and zeta potential showed that the free amino group content on BSF increased from 125.1 ± 1.2 µmol/mL to 153.5 ± 2.2 µmol/mL, the isoelectric point increased from 3.68 to 8.82, and the zeta potential reversed from − 11.8 ± 0.1 mV to + 12.4 ± 0.3 mV after PEI grafting. Positively charged CBSF could package pDNA to form spherical CBSF/pDNA complexes. In vitro, human lung adenocarcinoma A549 cells and human embryonic lung fibroblast WI-38 cells were transfected with CBSF/pDNA complexes. Confocal laser scanning microscopy analysis and flow cytometry tests showed that CBSF/pDNA complexes can effectively transfect A549 cells, and the transfection efficiency was higher than that of 25 kDa PEI/pDNA complexes. CCK-8 assay results showed that CBSF/pDNA complexes significantly inhibited the proliferation of A549 cells but had no significant effect on WI-38 cells and exhibited lower cytotoxicity to WI-38 cells than 25 kDa PEI. Therefore, a gene delivery system, constructed with the low-molecular-weight PEI-modified silk fibroin protein and the ING4-IL-24 double gene coexpression plasmid has potential applications in gene therapy for lung cancer.

## 1. Introduction

Lung cancer has become a leading cause of cancer-related deaths worldwide due to its high incidence, strong aggressiveness, late diagnosis, and lack of effective treatments [1]. Chemotherapy, surgical resection, and radiotherapy can be used to treat lung cancer, but these therapies have limited efficacy in treating advanced cancer and drug-resistant tumors [2]. Gene therapy involves using oligonucleotides to specifically target and regulate the abnormal genetic expression related to cancer development to cancer cells, which can significantly reduce the systemic cytotoxicity of cancer patients, so gene therapy was considered to be an efficient and promising cancer treatment method [3]. One of the key challenges for lung cancer gene therapy is to find therapeutic genes with remarkable curative effects and gene delivery carriers with high efficiency and low toxicity.

Growth inhibitory factor inhibitor of growth 4 (ING4), as an intracellular tumor growth inhibitory factor, can significantly inhibit the growth of tumor cells inducing cell cycle changes and cell apoptosis [4]. ING4 can inhibit the transcriptional activity of nuclear factor NF-κB and hypoxia-inducible factor HIF-1α to inhibit tumor angiogenesis, which has been revealed to have a suppressive role in various cancers, such as lung cancer, hepatocellular carcinoma, glioma, breast cancer, colon cancer, and ovarian carcinoma [5,6]. Interleukin-24 (IL-24) is a membrane receptor-mediated tumor growth inhibitory factor that inhibits the expression of the transcription factor GLI1 in lung cancer cells by inhibiting the Akt-mTOR and SDF-1/CXCR4 signaling axes, thereby inducing DNA damage in lung cancer cells and leading to cell death [7]. IL-24 can also indirectly inhibit angiogenesis by inhibiting the expression of PI3K/Akt signal, vascular endothelial growth factor VEGF, and transforming growth factor TGF-TGF, which play important roles in angiogenesis of lung cancer cells [8]. In addition, IL-24 shows special antitumor activity and can kill nearby tumor cells without toxicity to normal cells. Tumor cells often contain multiple genetic abnormalities, which limit the efficacy of a single gene-mediated cancer therapy [9]. The coexpression of the ING4 and IL-24 double genes can selectively inhibit tumor angiogenesis and the growth of tumor cells through pathways both inside and outside of the cells.

Another major challenge for lung cancer gene therapy is to find a gene delivery vector with high efficiency and low toxicity. Gene delivery vectors include viral vectors and non-viral vectors. Viral vectors have the advantage of high transfection efficiency, but they have the potential risk of carcinogenicity and immunogenicity [10]. In contrast, non-viral vectors, such as polyethylenimine (PEI), polylysine, cationic lipidosomes, and polysaccharides, have the advantages of having a large DNA delivery capacity and low immunogenicity [11]. High-molecular-weight polyethylenimine (25 kDa) is the “gold” standard for non-viral vectors due to its high transfection efficiency in different cell lines, but it shows significant cytotoxicity at the initial stage of treatment [12]. In contrast, low-molecular-weight PEI (1.8 kDa) shows lower cytotoxicity. However, the transfection efficiency is lower than that of high-molecular-weight PEI [13]. In order to improve the transfection efficiency of low-molecular-weight PEI, a variety of methods are used to modify PEI. For example, PEI conjugated with silica remarkably enhanced EGFP-N1 gene expression in murine neuroblastoma cells up to 38 fold compared to PEI 25 kDa [14]. However, their safety is still a severe challenge for in vivo applications because they are nondegradable. Biodegradable polymers, such as polyethylene glycol [15], polylactic acid [16], gelatin [17], and hyaluronic acid [18], are also used to modify PEI to minimize its cytotoxicity. However, polyethylene glycol and polylactic acid shield the positive charge of PEI, resulting in a decrease in transfection efficiency [19]. Gelatin and hyaluronic acid lack sufficient mechanical strength, and the fast degradation rate can easily lead to the rapid release of the target gene [20,21]. It is necessary to find biodegradable polymers to modify PEI in order to prepare gene carriers that can stably encapsulate the target gene, improve transfection efficiency, and reduce cytotoxicity.

Silk fibroin from *Bombyx mori* has been used as a tissue engineering scaffold and a delivery vehicle for biologically active molecules because of its unique combination of biocompatibility, controllable biodegradability, and mechanical stability [22,23]. When silk fibroin is used as a gene carrier, not only can the structural hierarchy, composition, and crystallinity be modified to achieve the designed release behavior without affecting the release of the target gene, but it also shows resistance capacity to DNA enzymes [24]. It can also protect DNA from potentially unstable conditions such as temperature and ultraviolet radiation [25]. In addition, silk fibroin can be modified through the carboxyl group of glutamic acid and aspartic acid, the phenolic hydroxyl group of tyrosine and the amino group of arginine, which improve the application of silk fibroin as a gene carrier [26]. In our previous study, we used spermine to modify *Bombyx mori* silk fibroin (BSF) [27]. Coating PEI/pDNA complexes with modified silk fibroin not only effectively shields the excessive positive charge on the surface of the PEI/pDNA complexes but also shows lower cytotoxicity and higher transfection efficiency than the PEI/pDNA complexes. BSF showed great potential as a gene delivery carrier.

In this study, BSF modified with low-molecular-weight PEI is used to encapsulate ING4 and IL-24 double gene coexpression plasmids to establish a high-efficiency and low-toxicity gene delivery carrier against lung cancer cells. We hypothesized that PEI can be grafted onto the side chains of BSF and that cationized *Bombyx mori* silk fibroin (CBSF) can be obtained. The positively charged silk fibroin can effectively package the ING4-IL-24 double gene coexpression plasmid pDNA to obtain CBSF/pDNA complexes. The complexes not only overcome the shortcomings of high-molecular-weight PEI with strong side effects but can also effectively transfect lung cancer cells and inhibit the growth of lung cancer cells and have no obvious cytotoxicity to normal cells. Based on this hypothesis, the carboxyl group of silk fibroin was activated first by using 1-ethyl-3- (3-dimethylaminopropyl) carbodiimide hydrochloride (EDC) to couple with the amino group of PEI to form CBSF. The zeta potential, trinitrobenzene sulfonic acid (TNBS), isoelectric points (pI), Fourier transform infrared (FTIR), and nuclear magnetism (^1^H-NMR) were used to characterize CBSF. Then, based on the electrostatic interaction, CBSF could wrap and compress pDNA to form CBSF/pDNA complexes. Agarose gel electrophoresis was used to detect the ability of CBSF to package pDNA. Zeta potential and scanning electron microscopy (SEM) were used to characterize the surface charge, particle size, and morphology of the composite. Finally, the CBSF/pDNA complexes were transfected into human embryonic lung adenocarcinoma cells A549 and human embryonic lung fibroblasts WI-38 in vitro. Confocal laser scanning microscopy (CLSM) observed the cell morphology and transfection effect. The cell counting kit-8 (CCK-8) method detected the effect of the complexes on cell proliferation and flow cytometry detected the cell transfection efficiency.

## 2. Materials and Methods

### 2.1. Preparation of Silk Fibroin Solution

*Bombyx mori* raw silks were degummed following a previously described procedure [28]. Briefly, the silk fibers (Nantong, China) were boiled three times in 0.05 wt% Na_2_CO_3_ aqueous solution for 30 min to remove sericin and dried at 60 °C after thorough rinsing with distilled water for subsequent experiments. The extracted fibers were dissolved in a ternary solvent system composed of calcium chloride, ethanol, and water (1:2:8 molar ratio) at 70 ± 2 °C for 1 h. After being completely cooled, the mixed solution was dialyzed (MWCO, 8–14 kDa) in deionized water for 3 days to obtain the regenerated BSF solution. The BSF solution was centrifuged (Heraeus PICO17, Thermo Scientific Company, Germany) at 10,000 rpm for 5 min and then stored in a 4 °C refrigerator.

### 2.2. Preparation of Cationized Silk Fibroin Solution

Fifty milliliters of the BSF solution (1.0 wt%) was cooled to 0~2 °C in an ice bath. Then, polyethylenimine (PEI, MW 1800 Da, Sigma-Aldrich) solution, which accounted for 0.1, 0.5, 1.0, 2.0, 4.0, 6.0, 8.0 and 10.0 wt% of the BSF weight in solution, was added. After adjusting the pH value to 7.2~7.5 with 0.1 M 2-(N-morpholino)ethanesulfonic acid (MES, Sigma-Aldrich) solution, N-hydroxysuccinimide (NHS, Sigma-Aldrich) was added to the mixture to account for 10 wt% of the weight of BSF in solution. After the pH value was stabilized, the pH of solution was adjusted to 5.0~6.0 by adding 0.1 M MES solution. Then, 1-ethyl-3-(3-dimethylaminopropyl) carbodiimide hydrochloride (EDC, Sigma-Aldrich) accounting for 20 wt% of the weight of BSF in solution was added. The reaction lasted for 6 h in an ice bath, and finally, the solution was incubated in a 4 °C refrigerator overnight. The mixed solutions were dialyzed with deionized water for 3 days. After centrifugation at 10,000 rpm for 5 min, the CBSF solution was obtained.

### 2.3. Characterization of CBSF

BSF and CBSF solutions were diluted with distilled water to 0.1 wt% for zeta potential measurement. To measure the potentials of the liquids, 1 mL sample solutions were taken and tested in a Zetasizer Nano ZS90 (Malvern Zetasizer Nano ZS90; Malvern Instruments, Malvern, UK) at 25 °C. Each group of samples was repeated three times.

To adjust the pH range of the solution from 3 to 10, 0.1 M hydrochloric acid and 0.1 M sodium hydroxide were used. Then the zeta potential values of BSF and CBSF at different pH values were measured to determine the pI value.

The content of free amino groups was determined by the trinitrobenzene sulfonic acid (TNBS) method [29]. Glycine was used as a standard. Standard solutions (20 µmol/mL, 1 mL) and test samples (0.5 mg/mL, 1 mL) were prepared. Each solution was equalized at 40 °C for 30 min after 1 mL of 4.0 wt% NaHCO_3_ buffer solution was added. Then, 1 mL of 0.05 wt% TNBS aqueous solution was added to the mixture. The reaction continued for 2 h at 40 °C. Finally, 1 mL of 1 M HCl was added. An ultraviolet spectrophotometer (UV-3600; Shimadzu, Tokyo, Japan) was used to measure the absorbance at 340 nm. The sample absorbance readings were changed to reflect the free amino group content using a standard curve obtained with glycine.

PEI, BSF and CBSF solids were obtained by freezing the solution at −80 °C for 6 h and then freeze-drying in a Virtis Genesis 25-LE lyophilizer for 48 h. The dry solids of PEI, BSF and CBSF were cut into tiny particles and screened with a 600 mesh sieve. Approximately 1 mg of particles was pressed into a pellet with 200 mg of potassium bromide. FTIR analysis was performed with a Nicolet 5700 spectrometer (Thermo Company, Madison, WI, USA). The measurements were taken in the range of 500–4000 cm^−1^.

Approximately 5 mg BSF, CBSF and PEI freeze-dried powders were dissolved in 400 µL of D_2_O, respectively. The solution was then loaded into a nuclear magnetic tube and the ^1^H-NMR spectra of the samples were measured using a superconducting nuclear magnetic resonance spectrometer (Avance III HD 400 MHz, Brucker, USA).

### 2.4. Construction of Recombinant Plasmids Coexpressing ING4 and IL-24 Double Genes

The construction method of the ING4-IL-24 double-gene coexpression plasmid containing green fluorescent protein (GFP) gene has been described previously [30]. Briefly, the ING4 and IL-24 fragments were amplified by polymerase chain reaction (PCR) using the pADTRACK-CMV-ING4 or pADTRACK-CMV-IL-24 plasmid as templates and combined with specific primers for ING4 or IL-24. The ING4 and IL-24 fragments were cloned into PADTrack-CMV-polyA + promoter transfer plasmids encoding the green fluorescent protein (GFP) gene at the BGL II, Sal I, Xhol I, and Xbal I sites, respectively. Then, the ING4-IL-24 double gene coexpression plasmid was propagated in Escherichia coli DH5α cells (Invitrogen, Carlsbad, CA, USA), and the ultrapure, endotoxin-free plasmid DNA was prepared using a QIA filtration kit (Qiagen, Chatsworth, CA, USA). The plasmid concentration was 100 ng/µL, which was measured by ultraviolet absorbance (Thermo Fisher Scientific, Waltham, MA, USA).

### 2.5. Preparation of CBSF/pDNA and PEI/pDNA Complexes

Both CBSF and 25 kDa PEI solutions were diluted to 1 µg/µL with deionized water. The CBSF/pDNA complex with a ratio of 4/2 was obtained by adding 4 µL of CBSF into a 20 µL of pDNA solution and then swirling for 30 min. The same method can also obtain CBSF/pDNA complexes with ratios of 8/2, 16/2, 32/2, 64/2, 128/2, 196/2, 256/2, and a 25 kDa PEI/pDNA complex with the ratio of 10/2.

### 2.6. Gel Retardation Assay

Agarose gel electrophoresis was used to analyze the packaging ability of CBSF and 25 kDa PEI to pDNA. The complex suspensions were loaded onto a 1.0 wt% agarose gel containing ethidium bromide (0.5 µg/mL) and run in Tris-acetate buffer at 100 V for 30 min. Finally, the results were observed by irradiation under UV light.

### 2.7. Characterization of CBSF/pDNA Complexes

The suspensions of CBSF/pDNA and PEI/pDNA complexes were tested in triplicate for particle size and surface charge using a zeta potential analyzer at 25 °C.

The composite solution was dripped on a silicon wafer. The liquid was blown dry with nitrogen and then sprayed with gold for 90 s. The surface morphology of the composite was observed under a scanning electron microscope (SEM, Hitachi S-4800, Hitachi Ltd., Tokyo, Japan).

### 2.8. In Vitro Gene Transfection

Human lung adenocarcinoma A549 and human embryonic lung fibroblast WI-38 were purchased from ATCC (Manassas, VA, USA). A549 and WI-38 cells were seeded on cell culture dishes (NEST, Lot No 2013008) at a density of 1 × 10^5^ and cultured in Dulbecco’s modified Eagle’s medium (DMEM, Gibco, USA), supplemented with 10% fetal calf serum (FBS, Gibco, USA) and 1% antibiotics (penicillin and streptomycin) at 37 °C using a humidified 5% CO_2_ incubator. Under microscopic visualization, when cells reached 85–90% confluence with normal morphology, they were washed with phosphate buffered saline (PBS) and placed in serum-free DMEM for 2 h. Then, after the cells were washed with PBS, binary complexes containing 2 μg of pDNA were diluted in 0.5 mL of DMEM and added dropwise to the cells. After 4 h of incubation, the medium was replaced by DMEM culture medium (10% FBS and 1% antibiotics) and continued to incubate for 24 h. The expression of green fluorescent protein in cells was observed by laser confocal microscope (CLSM, FV1000, Olympus, Japan) at 200 times.

### 2.9. Evaluation of Cell Viability

Cell viability was carried out using the CCK-8 assay. A549 cells and WI-38 cells were seeded in 96-well plates (TCP, Corning Inc., New York, NY, USA) 24 h prior to adding the complexes. When approximately 80% confluence of cells was achieved, the cells were incubated with CBSF/pDNA complexes in fresh serum-free DMEM. After 4 h of incubation, the media was replaced with 200 μL of DMEM culture medium (10% FBS and 1% antibiotics) for 24 h. Then CCK-8 (40 μL/well) and DMEM (400 μL/well) were added to the plate, and the cells were incubated for 2 h at 37 °C. The absorbance was recorded at 450nm by using a microplate reader (Synergy HT, BioTek Instruments, USA). Cells without incubated complexes were used as a control. Cell viability was calculated from the relation of absorbance, i.e., (Asample/Acontrol)*100 and plotted as a percentage of viability. Each experiment was performed in triplicate and repeated at least three times.

### 2.10. Transfection Efficiency Evaluation

The transfection efficiency of the complexes was measured by flow cytometry (FC500, Beckman-Coulter, USA). A549 cells were seeded at a density of 1 × 10^6^ cell/well in 6-well plates. The transfected method was as described above. The cells were trypsinized, washed, and resuspended in PBS. The expression levels of GFP were determined by flow cytometry (FC500, Beckman-Coulter, USA) within 30 min.

### 2.11. Statistical Analysis

All data are listed as means ± standard deviation. Statistical analysis was performed using one-way analysis of variance (*t*-test). Differences at *p* < 0.05 are considered statistically significant.

## 3. Results

### 3.1. Characterization of CBSF

**Figure 1:**
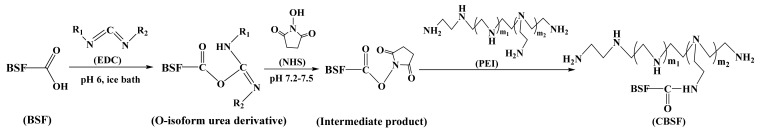
Schematic diagram of CBSF synthesis.

The synthesis principle of CBSF is shown in Figure 1. The carbon-nitrogen double bond (–N=C–) on the water-soluble EDC can couple with the carboxyl groups of aspartic acid and glutamic acid in silk fibroin to form unstable O-isouryl urea derivatives. Subsequently, the free hydroxyl (–OH) on the NHS reacted with the unstable derivative to form a stable intermediate product. The intermediate product further reacted with the free amino group in PEI to form a new amide bond, and finally CBSF was obtained.

**Figure 2:**
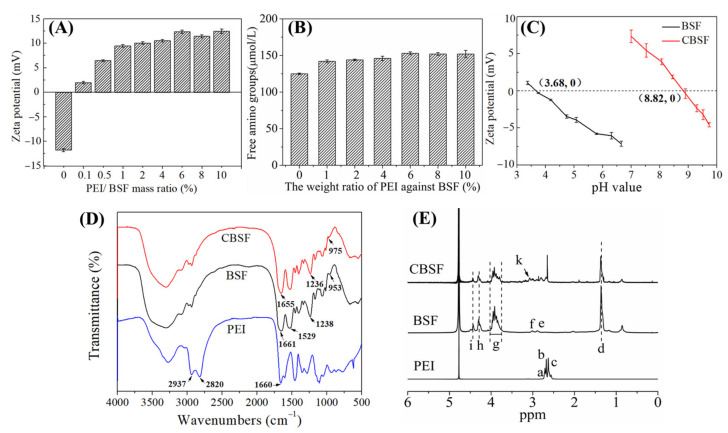
The characteristics of CBSF. (**A**) Zeta potentials of CBSF at different weight ratios of PEI with respect to BSF. (**B**) TNBS assay for the determination of free amino groups in PEI-modified BSF. (**C**) Isoelectric point values of BSF and CBSF (modified with 6.0 wt% PEI against BSF). (**D**) FTIR spectra of PEI, BSF, and CBSF. (**E**) ^1^H-NMR spectra of PEI, BSF, and CBSF. a: ~2.71 ppm; b: ~2.68 ppm; c: ~2.62 ppm; d: ~1.24 ppm; e: ~2.78 ppm; f: ~2.91 ppm; g: 3.63~3.91 ppm; h: ~4.18 ppm; i: ~4.32 ppm; k: ~3.12 ppm. Each value represents the mean ± SD of three separate experiments (n = 3 per experiment).

Figure 2A shows the zeta potential changes of the silk fibroin after reacting with different proportions of PEI. Under neutral conditions, the carboxyl groups on the side chains of aspartic acid and glutamic acid of silk fibroin easily lost H^+^ and ionized into –COO–, causing the pure silk fibroin to become negatively charged and its zeta potential was approximately −11.8 ± 0.1 mV. When reacting with 0.1 wt% PEI, the zeta potential of silk fibroin turned from negative to positive, which was +1.9 ± 0.2 mV. With the increasing of PEI from 0.5 wt% to 4.0 wt%, the zeta potentials increased from +6.5 ± 0.2 mV to +10.5 ± 0.3 mV. When PEI accounting for 6.0% of the silk fibroin mass was added, the zeta potential of CBSF reached +12.4 ± 0.3 mV. As the mass ratios of PEI/BSF continued to increase, there was no significant increase in the zeta potential of CBSF.

The content of free amino groups in BSF modified by PEI is shown in Figure 2B. The content of free amino groups in unmodified BSF was 125.1 ± 1.2 µmol/mL. As the content of PEI increased from 1.0 wt% to 6.0 wt%, the concentration of free amino groups in BSF increased from 142.6 ± 2.4 µmol/mL to 153.5 ± 2.2 µmol/mL. However, there was no significant increase in free amino content after the addition of 8.0 wt% and 10.0 wt% PEI, indicating that BSF modified by 6.0 wt% PEI could maximize the concentration of free amino groups in the side chains.

The pI values of BSF and CBSF were measured by potentiometric titration at pH 3~10, as shown in Figure 2C. The pI of the original BSF was approximately 3.68. After modification by 6.0 wt% PEI with respect to the weight of BSF, the pI of CBSF significantly increased to approximately 8.82, suggesting that the negatively charged carboxyl groups in the side chains of BSF were consumed in the amidation reaction, exposing a large number of amino groups on the surface of BSF and changing the surface charge of BSF from negative to positive. The results demonstrated that BSF could be endowed effectively with a large amount of positive charge by modification with 6.0 wt% PEI.

The FTIR spectra of PEI, BSF, and CBSF are shown in Figure 2D. The absorptions at 2937 cm^−1^ and 2820 cm^−1^ represent the symmetrical and asymmetrical stretching vibration absorption of –CH_2_ in PEI, respectively. The in-plane bending vibration of the primary amino group (–NH_2_) appeared at 1660 cm^−1^ [31,32]. The characteristic absorptions of BSF appeared at 1661 cm^−1^ (C=O stretching vibration, amide I), 1529 cm^−1^ (N–H bending vibration and C–H stretching vibration, amide II), and 1239 cm^−1^ (C–N stretching vibration and N–H bending vibration, amide III), and the absorption at 953 cm^−1^ represented the out of plane bending vibration of O–H in carboxyl groups [33,34]. After the modification of BSF with PEI, the characteristic absorptions of BSF at 1661 cm^−1^, 1238 cm^−1^, and 953 cm^−1^ shifted to 1655 cm^−1^, 1236 cm^−1^, and 975 cm^−1^ in CBSF, respectively, suggesting that the –COOH on the side chain of BSF reacted with the –NH_2_ of PEI to form a new amide bond.

Figure 2E shows the ^1^H-NMR spectra of PEI, BSF, and CBSF. In the ^1^H-NMR spectra of PEI, δ 2.31~2.85 ppm (points a, b, and c) was the chemical shift corresponding to methylene protons in the –NH–CH_2_–CH_2_– group of the PEI monomer [35]. In the ^1^H-NMR spectra of BSF, the d point (δ ~ 1.24 ppm), e point (δ ~ 2.78 ppm), and f point (δ ~ 2.91 ppm) were the chemical shifts of the –CH–NH_2_CH_3_ proton in Ala, –CHNH_2_–CH_2_– proton in Tyr and –CHNH_2_–CH_2_– proton in Asp, respectively, and the δ 3.63~3.91 ppm g point was the chemical shift of the NH_2_–CH_2_– proton in Gly and the NH_2_–CH_2_–CH– proton in Ser. The chemical shift of –CH–NH_2_CH_3_ protons in Ala appeared at the h point (δ ~ 4.18 ppm) and δ ~ 4.32 ppm at the i point was the chemical shift of the –CH–NH_2_OH proton in Ser [36]. In the ^1^H-NMR spectrum of CBSF, a new proton peak appeared at the k point (δ ~ 3.12 ppm), which corresponded to the chemical shift of the methylene proton close to the amide bond in the –CONHCH_2_CH_2_NH– group [37], indicating that –COOH on the side chains of BSF reacted with the –NH_2_ of PEI to form an amide bond. The above results demonstrated that BSF was effectively modified by PEI. CASF obtained by modification of ASF with 6.0% PEI was used in subsequent experiments.

### 3.2. Characterization of CBSF

**Figure 3:**
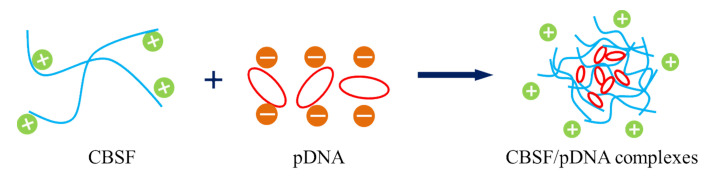
Schematic illustration of the formation of the CBSF/pDNA complexes.

**Figure 4:**
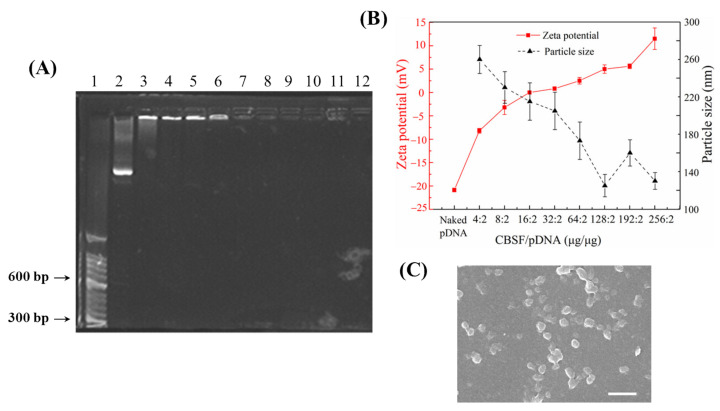
(**A**) Agarose gel electrophoresis of naked pDNA, CBSF/pDNA complexes and PEI/pDNA complexes. Lane 1: marker; lane 2: naked pDNA; lanes 3–10: CBSF/pDNA complexes at *w*/*w* ratios of 4/2, 8/2, 16/2, 32/2, 64/2, 128/2, 196/2, and 256/2; lane 11: 1.8 kDa PEI/pDNA complex at a *w*/*w* ratio of 10/3; lane 12: 25 kDa PEI/pDNA complex at a *w*/*w* ratio of 10/2. (**B**) Zeta potentials and particle sizes of complexes at different CBSF/pDNA weight ratios. (**C**) Scanning electron microscopy image of the CBSF/pDNA complex at a *w*/*w* ratio of 256/2. Scale bar: 400 nm.

The negatively charged pDNA was packaged by positively charged CBSF via electrostatic interactions to form CBSF/pDNA complexes (Figure 3). Agarose gel electrophoresis was performed for CBSF/pDNA complexes at different mass ratios (Figure 4A) to assess the ability of CBSF to encapsulate pDNA. Marker (lane 1) and naked pDNA (lane 2) showed different fluorescent bands. When the mass ratios of CBSF/pDNA were 4/2, 8/2, 16/2, and 32/2 (Lanes 3, 4, 5, and 6), clear fluorescent bands of pDNA could be seen in the figure, but the migration of pDNA was blocked, suggesting that CBSF could encapsulate pDNA. When the mass ratios of CBSF/pDNA were 64/2, 128/2, 192/2, and 256/2 (lanes 7, 8, 9, and 10), the migrations of pDNA were completely blocked, suggesting that when the mass ratios of CBSF to pDNA were greater than 64/2, CBSF could completely encapsulate and compress pDNA through electrostatic interactions. At this time, the blocking band of CBSF/pDNA was similar to that of 25kDa PEI/pDNA (lane 12), indicating that the encapsulation ability of CBSF to pDNA was similar to that of 25 kDa PEI.

Since the surface charges and sizes of the complexes would affect cell uptake to the complexes, the zeta potentials and particle sizes of CBSF/pDNA complexes at different mass ratios were tested [38], as shown in Figure 4B. The zeta potential of naked pDNA was −20.9 ± 0.4 mV. The zeta potential value of the CBSF/pDNA complex at a weight ratio of 4/2 was −8.2 ± 0.5 mV, and the particle size was ~260 nm. As the mass ratio of CBSF/pDNA reached 32/2, the zeta potential of the complex reversed from negative to positive, which was +0.8 ± 0.4 mV, and the particle size decreased to ~205 nm. When the mass ratio of CBSF/pDNA increased from 64/2 to 256/2, the zeta potential increased from +2.5 ± 0.7 mV to +11.5 ± 2.5 mV, and the particle size decreased from ~170 nm to ~120 nm. The morphology of the CBSF/pDNA (256/2) complex was observed by SEM (Figure 4C). The pDNA was compressed and wrapped by CBSF to form a spherical complex with a diameter of approximately 100–200 nm (Figure 4C).

### 3.3. In Vitro Transfection of A549 and WI-38 Cells with CBSF/pDNA Complexes

**Figure 5:**
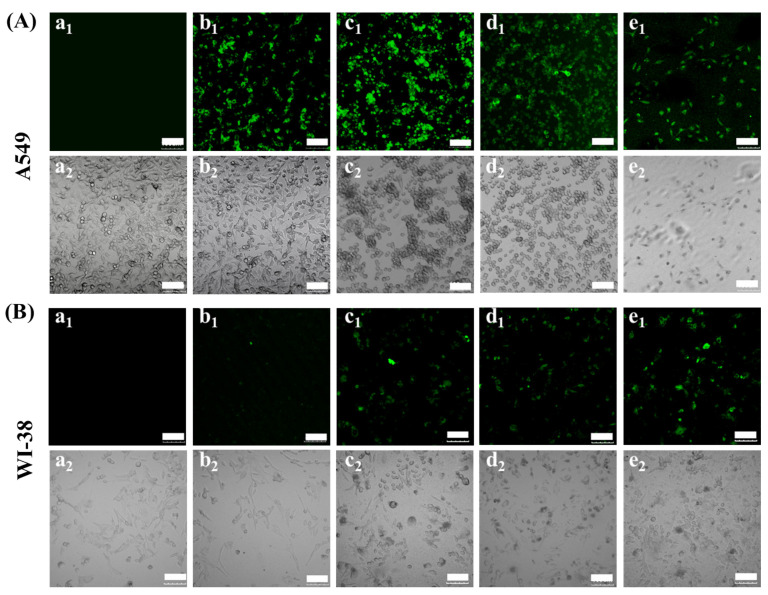
(**A**) Fluorescent micrographs of A549 cells transfected with different formulations. (**B**) Fluorescent micrographs of WI-38 cells transfected with different formulations. (**a**): Untreated cells; (**b**–**d**) represent CBSF/pDNA complexes with ratios of 64/2, 128/2, and 256/2, respectively; (**e**): A 25 kDa PEI/pDNA complex at a *w*/*w* ratio of 10/2. (**a_1_**–**e_1_**) and (**a_2_**–**e_2_**) denote the fluorescent field and bright field, respectively. Scale bar: 50 µm.

A549 cells were transfected with different mass ratios of CBSF/pDNA complexes in vitro, and the expression of green fluorescent protein in cells and cell morphology were observed by CLSM, as shown in Figure 5A. The cells without the complexes did not show green fluorescence (Figure 5A(a_1_)). After the complex formed by 64 µg of CBSF encapsulating 2 µg of pDNA was cocultured with cells for 24 h, the cells showed green fluorescence (Figure 5A(b_1_)), indicating that the CBSF/pDNA complex could transfect A549 cells. When the CBSF/pDNA mass ratio increased to 128/2, the number of cells expressing green fluorescence increased and the fluorescence intensity increased (Figure 5A(c_1_)). However, when the ratio of CBSF/pDNA continued to increase to 256/2 (Figure 5A(d_1_)), the fluorescence intensity decreased, but it was similar to that of 25 kDa PEI/pDNA (Figure 5A(d_1_,e_1_)), indicating that the transfection efficiency of CBSF/pDNA (256/2) transfected A549 cells was similar to that of 25 kDa PEI/pDNA (10/2). From the bright field figures, it can be seen that the untransfected A549 cells were evenly distributed with a fusiform shape (Figure 5A(a_2_)). When cells were cocultured with the CBSF/pDNA (64/2) complex for 24 h, a few cells became round (Figure 5A(b_2_)). When the ratios of CBSF/pDNA complexes increased to 128/2 and 256/2, most cells were rounded and partially aggregated (Figure 5A(c_2_, d_2_)), indicating that the cell viability of A549 cells decreased. Compared with A549 cells transfected with the 25 kDa/pDNA (10/2) complex (Figure 5A(e_2_)), the circular cell density was higher after transfection with CBSF/pDNA complexes (128/2 and 256/2), showing that CBSF/pDNA (128/2 and 256/2) complexes had a stronger inhibitory effect on the proliferation of A549 cells.

WI-38 cells were selected for coculture with different mass ratios of CBSF/pDNA complexes for 24 h. The CLSM images are shown in Figure 5B. Cells without complex coculture did not show green fluorescence (Figure 5B(a_1_)). When WI-38 cells were cocultured with the CBSF/pDNA complex at a ratio of 64/2, some cells showed weak green fluorescence (Figure 5B(b_1_)). As the ratios of CBSF/pDNA increased to 128/2 and 256/2, the number of cells expressing green fluorescence increased, and the fluorescence intensities were significantly enhanced (Figure 5B(c_1_,d_1_)), but lower than those of cells cocultured with the 25 kDa/pDNA (10/2) complex (Figure 5B(e_1_)). From the bright field diagram, the untransfected WI-38 cells were evenly distributed and fusiform (Figure 5B(a_2_)). The addition of the CBSF/pDNA complex at a ratio of 64/2 had no significant effect on cell morphology (Figure 5B(b_2_)). When the ratios of CBSF/pDNA complexes were 128/2 and 256/2, partial cells were still similar to the morphology of untransfected cells although local cells became round (Figure 5B(c_2_, d_2_)), while most cells became round after adding 25 kDa/pDNA (10/2) complex to the WI-38 cells (Figure 5B(e_2_)). The results showed that CBSF as a gene carrier reduced cytotoxicity to normal cells compared with 25 kDa PEI.

### 3.4. Transfection Efficiency of Complexes Transfect A549 Cells

**Figure 6:**
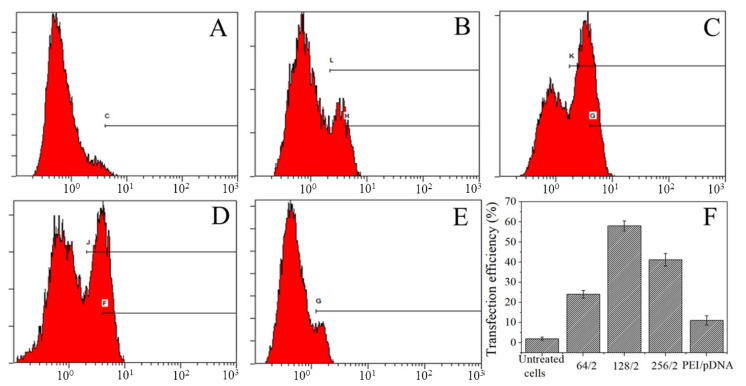
Transfection efficiency of A549 cells transfected with CBSF/pDNA complexes at different mass ratios for 24 h. (**A**): untreated cells; (**B**–**D**) represent CBSF/pDNA complexes with ratios of 64/2, 128/2, and 256/2, respectively. (**E**): A 25 kDa PEI/pDNA complex at a *w*/*w* ratio of 10/2. (**F**): Transfection efficiency of two complexes at different mass ratios. Each value represents the mean ± SD of three separate experiments.

Flow cytometry was used to detect the transfection efficiency after the A549 cells were cocultured with different mass ratios of complexes for 24 h, as shown in Figure 6. The cell transfection efficiency without complexes was 1.9%. While the ratios of CBSF/pDNA complexes were 64/2, 128/2, and 256/2, the transfection efficiencies of A549 cells were 24.1%, 58.2%, and 41.2%, respectively, which were all higher than 11.2% of 25 kDa PEI/pDNA, indicating that CBSF/pDNA complexes could transfect A549 cells effectively and express green fluorescent proteins.

### 3.5. Effects of CBSF/pDNA Complexes on the Proliferation of A549 and WI-38 Cells

**Figure 7:**
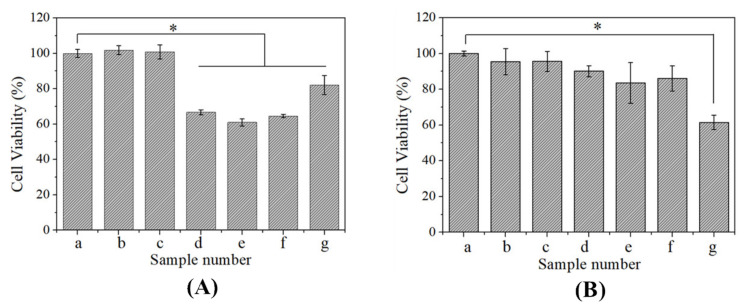
Viability of (**A**) A549 cells and (**B**) WI-38 cells after culturing with CBSF/pDNA complexes at predetermined weight ratios. **a**: Blank control; **b**: 2 µg pDNA; **c**: 128 µg CBSF; **d**–**f**: CBSF/pDNA complexes at *w*/*w* ratios of 64/2, 128/2, and 256/2, respectively. **g** A 25 kDa PEI/pDNA complex at a *w*/*w* ratio of 10/2. Each value represents the mean ± SD of three separate experiments, n = 3, * *p* < 0.05.

As shown in Figure 7A, the cell viabilities of A549 cells and WI-38 cells transfected with different mass ratios of CBSF/pDNA complexes were measured by CCK-8 assay. The cell viability of A549 cells cultured with naked pDNA and CBSF was 101.8 ± 2.5 and 100.9±4.0% respectively, indicating that pDNA and CBSF had no significant effects on the proliferation of A549 cells. After adding the CBSF/pDNA complexes at ratios of 64/2, 128/2, and 256/2, the cell viability decreased to 66.7 ± 1.4%, 61.1 ± 2.1%, and 64.6 ± 1.2%, respectively, indicating that the addition of CBSF/pDNA complexes could significantly inhibit A549 cell proliferation (*p* < 0.05). The cell viability after adding CBSF/pDNA complexes was lower than that after adding the 25 kDa PEI/pDNA complex (82.1 ± 5.4%, *p* < 0.05), suggesting that the CBSF/pDNA complexes more significantly inhibited the proliferation of cancer cells compared with the 25 kDa PEI/pDNA complex.

As shown in Figure 7B, when 2 µg of pDNA and 128 µg of CBSF were added, the cell viabilities of WI-38 cells were 95.5 ± 7.3% and 95.6 ± 5.6%, respectively, indicating that pDNA and CBSF did not affect the proliferation of WI-38 cells. When the ratios of CBSF/pDNA complexes were 64/2, 128/2, and 256/2, the cell viability decreased to 90.1 ± 3.1%, 83.6 ± 11.4%, and 86.1 ± 7.0%, respectively. The values were higher than that of WI-38 cells transfected with the 25 kDa PEI/pDNA complex (61.5 ± 4.2%, *p* < 0.05), indicating that the CBSF/pDNA complexes showed lower cytotoxicity to normal cells than the 25 kDa PEI/pDNA complexes.

## 4. Discussion

Efficient transfection with minimal associated cytotoxicity and safety concerns are the key qualities of gene carriers in cancer gene therapy. In non-viral gene vectors, high molecular weight polyethylenimine (PEI, 25 kDa) is considered the “gold” standard for gene delivery because of the proton sponge effect and the ability to protect DNA from degradation by enzymes [15,27]. However, its high charge density would cause severe cytotoxicity [39]. In contrast, PEI with a low-molecular-weight showed low cytotoxicity, but its transfection efficiency was limited [40]. To overcome the contradiction between transfection and toxicity, in this study, we attempted to graft low-molecular-weight PEI with *Bombyx mori* silk fibroin protein, in order to improve the transfection efficiency of low-molecular-weight PEI while giving it lower cytotoxicity.

The glutamic acid and aspartic acid in the side chains of BSF contain carboxyl groups, which could react with the carbon-nitrogen double bond (–N=C–) in EDC to form O-isourylurea derivatives. In the presence of NHS, they were converted into amino-active sulfonyl NHS esters. Then, the product reacted with the free amine group in PEI to form a stable amide bond, and finally CBSF was obtained (Figure 2A). During this reaction, the carboxyl groups of BSF were consumed, while the amino group content on the surface of CBSF increased. Therefore, as the amount of added PEI increased, the content of free amino groups of CBSF and the zeta potential increased at the beginning of the reaction (Figure 2A,B)). When 6.0 wt% PEI was added, the amino group content increased to 153.5 ± 2.2 µmol/mL, and the zeta potential increased to +12.4 ± 0.3 mV. However, since the branched chain of PEI contained more protonated secondary amine and fulcrum amine groups, the carboxyl groups in BSF were quickly consumed. When the amount of added PEI was greater than 6.0 wt%, the carboxyl groups on the side chains of BSF were almost completely consumed, so the free amino group content and zeta potential in CBSF did not change significantly at this time. Compared with the BSF solution, the CBSF solution modified with 6.0 wt% PEI had reduced carboxyl groups and increased amino group content, which increased the pI from 3.68 to 8.82 (Figure 2D). It can be observed from the FTIR spectrum of CBSF that the characteristic absorption of C=O (1661 cm^−^^1^), C–N (1238 cm^−^^1^), and O–H (953 cm^−^^1^) in BSF were shifted to 1655 cm^−^^1^, 1236 cm^−^^1^, and 975 cm^−^^1^, respectively (Figure 2D). The changes could be attributed to the O=C–N in-plane bending vibration, which was caused by the reaction between –COOH in the side chains of BSF and –NH_2_ in PEI [27]. In addition, in the ^1^H-NMR spectrum, the methylene proton peak at δ~3.12 ppm corresponded to the formation of a new amide bond (Figure 2E). The above results indicated that PEI was grafted onto the side chains of BSF.

One key aspect of gene delivery is the packaging of genetic material into structures which can be efficiently transported into cells [41]. Therefore, the ability of non-viral vectors to bind nucleic acids is a prerequisite for gene delivery. Negatively charged BSF in solution was not able to encapsulate the negatively charged pDNA because of charge repulsion, so the migration of pDNA still occurred (Figure 4A, lane 2). BSF modified by PEI was positively charged and could encapsulate pDNA via electrostatic interactions to form CBSF/pDNA complexes (Figure 3). When the ratios of CBSF/pDNA were greater than 64/2, the encapsulation effect of CBSF on pDNA was similar to that of 25 kDa PEI (Figure 4A) and the zeta potentials of the complexes reversed to positive values (Figure 4B). The positive charge of the complexes can help to bind to the surface of the anionic cells and promote cell uptake of the complexes, thus improving the transfection efficiency [42]. However, cell uptake is also closely related to the size of the complexes [43]. In this study, CBSF could encapsulate pDNA into compact spherical CBSF/pDNA (256/2) complexes with a diameter of approximately 100–200 nm (Figure 4C) and nanoparticles of this size can be endocytosed by the cells [44].

In order to evaluate the transfection effectiveness of the CBSF/pDNA complexes in cancer cells and normal cells, CBSF/pDNA complexes with mass ratios of 64/2, 128/2, and 256/2 were cocultured with A549 and WI-38 cells. The 64/2 CBSF/pDNA complex showed a positive zeta potential (Figure 4B), it could adhere to the negatively charged A549 cell membrane surface through electrostatic interactions, and be further internalized by the cells for effective transfection, with a transfection efficiency of 24.1% (Figure 6b). Meanwhile, there was obvious expression of GFP in A549 cells (Figure 5A(b_1_)). Compared with the 64/2 CBSF/pDNA complex, the 128/2 CBSF/pDNA complex had more positive charges (Figure 4B) and showed stronger adhesion to the cell membrane, resulting in the transfection efficiency significantly increasing to 58.2% (Figure 6C) and the expression of GFP being enhanced (Figure 5A(c_1_)). In contrast, the transfection efficiency of the 256/2 CBSF/pDNA complex on A549 cells was reduced (Figure 6D), which may be because CBSF with a high mass ratio can form a strong condensation ability with pDNA, which made it difficult for cells to release pDNA from the complex after ingestion, leading to a reduced transfection efficiency [45]. Importantly, the transfection efficiency of the above proportions of CBSF/pDNA complexes was higher than that of 25kDa PEI/pDNA 11.05% (Figure 6E), indicating that CBSF had a higher transfection efficiency to cancer cells than 25kDa PEI. Moreover, the ING4-IL-24 double gene can inhibit the proliferation of cancer cells intracellularly and extracellularly [9]. The number of round A549 cells in the field of vision increased (Figure 5A(b_2_–d_2_)) and the cell viability of the A549 cells significantly decreased (Figure 7A) as the mass ratios of CBSF/pDNA increased. If CBSF was modified by ligands that specifically identify lung cancer cells (e.g., transferrin, transformation factor α, hyaluronic acid, folic acid, and peptides) and used to package pDNA, the complex is expected to further inhibit the proliferation of cancer cells [46,47]. These results suggested that CBSF, as a gene carrier, could effectively deliver the ING4-IL-24 double gene, thereby inhibiting the proliferation of A549 cells. For normal WI-38 cells, the CBSF/pDNA complexes could be transfected into cells, but showed lower GFP expression than cancer cells (Figure 5B(b_1_–d_1_)). This may be due to the fact that normal cells express different numbers of receptors on the surface of the cell membrane compared to cancer cells, which may affect the available binding sites to the complexes and the uptake of the complexes [28]. Different transfection efficiencies and GFP expression of the same gene vector in different cell lines have also been found in other studies [48]. These results illustrated that CBSF/pDNA complexes could effectively transfect A549 cells, reaching thetransfection efficiency higher than that of 25 kDa PEI/pDNA complexes, and inhibiting the proliferation of A549 cells.

Cytotoxicity is an important factor in evaluating the application of vectors in gene delivery. The cell morphology of WI-38 cells cocultured with the CBSF/pDNA complexes was similar to that of untransfected cells (Figure 5B(a_1_–d_1_)), with cell viability maintained at over 80% (Figure 7B). However, cells cocultured with 25kDa PEI/pDNA were almost round (Figure 5B(e_1_)), and cell viability was significantly decreased (Figure 7B). It is mainly due to the zeta potential of CBSF/pDNA complex being lower than 25kDa PEI/pDNA complex [27,49]. Therefore, compared with 25kDa PEI/pDNA, CBSF/pDNA significantly reduced the cytotoxicity to normal lung WI-38 cells. In our study, it can be observed that the CBSF/pDNA complex showed selective cytotoxicity and that the proliferation of A549 cells was significantly inhibited but there was no significant effect on WI-38 cells (Figure 7A,B). This might be derived from the expression of IL-24. A study showed that IL-24 inhibited the expression of the transcription factor GLI1, which is overexpressed in lung cancer cells, by inhibiting the Akt-mTOR and SDF-1/CXCR4 signaling axes, thereby inducing DNA damage in lung cancer cells and leading to cell death, however, the protein expression of GLI1 in normal cells did not change significantly [7]. Although the uptake of the complexes by cells has been identified, the expression of the ING4-IL-24 double gene after transfection of the complexes and the inhibitory effect on tumor cells still needs to be further explored. The results of this paper show that CBSF, as a gene carrier, can encapsulate the ING4-IL-24 double gene and further effectively transfect lung cancer A549 cells and significantly inhibit the proliferation of A549 cells, but has no obvious toxicity to normal WI-38 cells.

## 5. Conclusions

In this study, BSF was modified with low-molecular-weight (1.8 kDa) PEI. The obtained CBSF could encapsulate double gene coexpression plasmid DNA encoded by ING4 and IL-24 to form CBSF/pDNA complexes. The complexes were transfected into human lung adenocarcinoma cells A549 and human embryonic lung fibroblasts WI-38 in vitro. Zeta potentials, isoelectric points, the content of free amino groups, FTIR and ^1^H-NMR results showed that the amino groups in PEI reacted effectively with the carboxyl groups on the side chains of BSF, which made the surface charges of BSF change from negative to positive. Based on the electrostatic interaction, CBSF can package pDNA to form nanoscale spherical particles. When CBSF/pDNA complexes were transfected into A549 cells in vitro, the transfection efficiencies were higher than that of the 25 kDa PEI/pDNA complexes. In particular, the transfection efficiency reached approximately 58.2% when the CBSF/pDNA ratio of the complex was 128/2, which significantly inhibited the proliferation of A549 cells, and importantly, there was no significant change in WI-38 cell proliferation, and the toxicity of CBSF to WI-38 cells was significantly lower than that of 25 kDa PEI. CBSF may be used as a new gene carrier in clinical gene therapy.

## Data Availability

Data is contained within the article.
